# Clinical Efficacy and Post-Treatment Seromarkers Associated with the Risk of Hepatocellular Carcinoma among Chronic Hepatitis C Patients

**DOI:** 10.1038/s41598-017-02313-y

**Published:** 2017-06-16

**Authors:** Mei-Hsuan Lee, Chung-Feng Huang, Hsueh-Chou Lai, Chun-Yen Lin, Chia-Yen Dai, Chun-Jen Liu, Jing-Houng Wang, Jee-Fu Huang, Wen-Pang Su, Hung-Chih Yang, Kwong-Ming Kee, Ming-Lun Yeh, Po-Heng Chuang, Shih-Jer Hsu, Ching-I Huang, Jung-Ta Kao, Chieh-Chang Chen, Sheng-Hung Chen, Wen-Juei Jeng, Hwai-I Yang, Yong Yuan, Sheng-Nan Lu, I-Shyan Sheen, Chen-Hua Liu, Cheng-Yuan Peng, Jia-Horng Kao, Ming-Lung Yu, Wan-Long Chuang, Chien-Jen Chen

**Affiliations:** 1 Institute of Clinical Medicine, National Yang-Ming University, Taipei, Taiwan; 2 Hepatobiliary Division, Department of Internal Medicine, Kaohsiung Medical University, Kaohsiung, Taiwan; 3 Faculty of Internal Medicine, College of Medicine, Kaohsiung Medical University, Kaohsiung, Kaohsiung Taiwan; 4 Division of Hepatogastroenterology, Department of Internal Medicine, China Medical University Hospital, Taichung, Taiwan; 5 Department of Gastroenterology and Hepatology, Linkou Medical Center, Chang Gung Memorial Hospital, Kweishan, Taoyuan Taiwan; 6 College of Medicine, Chang Guang University, Kweishan, Taoyuan Taiwan; 7 Graduate Institute of Clinical Medicine, College of Medicine, Kaohsiung Medical University, Kaohsiung, Taiwan; 8 Department of Internal Medicine, National Taiwan University Hospital and National Taiwan University College of Medicine, Taipei, Taiwan; 9 Hepatitis Research Center, National Taiwan University Hospital, Taipei, Taiwan; 10 Graduate Institute of Clinical Medicine, National Taiwan University College of Medicine, Taipei, Taiwan; 11 Division of Hepato-Gastroenterology, Department of Internal Medicine, Kaohsiung Chang Gung Memorial Hospital and Chang Gung University College of Medicine, Kaohsiung, Taiwan; 12 Department of Microbiology, National Taiwan University College of Medicine, Taipei, Taiwan; 13 Department of Internal Medicine, National Taiwan University Hospital, Yun-Lin Branch, Yun-Lin, Taiwan; 14 School of Traditional Chinese Medicine, Chang Guang University, Kweishan, Taoyuan, Taiwan; 15 Genomics Research Center, Academia Sinica, Taipei, Taiwan; 16 Global Health Economics and Outcomes Research, Bristol Myers-Squibb, Princeton, NJ USA; 17 School of Medicine, China Medical University, Taichung, Taiwan; 18 Institute of Biomedical Sciences, National Sun Yat-Sen University, Kaohsiung, Taiwan; 19 Liver Center, Division of Gastroenterology, Massachusetts General Hospital, Harvard Medical School, Boston, MA USA; 20 Academia Sinica, Taipei, Taiwan

## Abstract

This follow-up study enrolled chronic hepatitis C patients to evaluate the treatment efficacy and to identify post-treatment seromarkers associated with risk of hepatocellular carcinoma (HCC) among patients with a sustained virological response (SVR) or nonsustained virological response (NSVR). A total of 4639 patients who received pegylated interferon and ribavirin during 2004–2013 were followed until December 2014. HCC was confirmed through health examinations and data linkage with a national database. A total of 233 HCC cases were reported after 26,163 person-years of follow-up, indicating an incidence of 8.9 per 1000 person-years: 6.9 for SVR and 21.6 for NSVR per 1000 person-years. The associated risk of HCC in patients with SVR was 0.37 (0.22–0.63) for those without cirrhosis and 0.54 (0.31–0.92) for those with cirrhosis compared with their respective counterparts with NSVR. Among patients with SVR, advanced age, male gender, cirrhosis, decreased platelet count, and increased aspartate aminotransferase and α-fetoprotein levels were associated with HCC (p < 0.001). The treatment of chronic hepatitis C patients before they developed cirrhosis showed a higher efficacy than did the treatment of those who had already developed cirrhosis. Patients with SVR may still have a risk of HCC and need to be regularly monitored.

## Introduction

The World Health Organisation estimates that more than 185 million people (2.8%) have been infected with hepatitis C virus (HCV) globally^1^, and approximately 50–60% of these HCV-infected people live in the western Pacific and southeast Asian regions^2^. According to previous estimations, HCV infection accounts for more than 25% of the worldwide hepatocellular carcinoma (HCC) occurrence^3^. Individuals with chronic hepatitis C infection may have an increased risk of developing end-stage liver disease and extrahepatic diseases^4, 5^.

In recent years, a highly effective, short treatment course of orally administered direct-acting antiviral agents has been approved^6, 7^. However, these expensive drugs are not affordable for all HCV-infected patients and for citizens from countries with limited healthcare resources. In many countries, pegylated interferon (Peg-IFN) and ribavirin remain the first-line regimen for chronic hepatitis C patients. Successful treatment is defined as a sustained virological response (SVR), which is the absence of viremia for 24 weeks after the cessation of treatment. In Asian countries, most individuals carry the favourable IL28B genotype, resulting in a superior response to interferon-based therapy compared with that observed among other ethnic populations^8^. The duration of required clinical follow-up for patients who have achieved SVR is still unclear. Therefore, investigating the predictors for the development of HCC among patients who have achieved treatment-induced RNA clearance is critical for future clinical monitoring. The identification of HCC predictors among nonresponders and patients with SVR will be helpful when prioritising patients to receive intensive care.

Interferon-based treatment has been documented to reduce the risk of liver transplantation, liver failure, HCC, and liver-related mortality^9–13^. Compared with nonresponders, patients with SVR showed normalized seromarkers in laboratory tests^14^ and obvious beneficial effects^14–19^, with a 70% decrease in the development of end-stage liver disease^18^ and 54–75% decrease in all-cause mortality^15, 16^. However, most of these studies recruited highly selected patients with advanced fibrosis or cirrhosis^13, 14, 16, 19^. The treatment efficacy in noncirrhotic patients who are still in the early clinical stages has rarely been evaluated. Infected patients are often unaware of their infection and therefore do not seek clinical care and treatment because liver disease progresses slowly with few symptoms. Because large-scale screening has been advocated to identify patients with a high risk of HCV infection, evaluating the treatment efficacy may provide information for future cost-effective estimations.

This long-term follow-up study evaluated the treatment efficacy in patients who used the conventional Peg-IFN and ribavirin. Additionally, we examined clinical predictors for the risk of HCC among chronic hepatitis C patients who received interferon-based antiviral therapy.

## Methods

We conducted a multicentre prospective study to investigate the HCC risk among chronic hepatitis C patients who received Peg-IFN and ribavirin. The study consisted of 4639 treatment-naive patients who received standard care in six medical centres located in northern, middle, and southern Taiwan during 2004–2014. All participants were aged 30 years or older; seropositive for HCV antibodies, with detectable HCV RNA; seronegative for hepatitis B surface antigen; seronegative for human immunodeficiency virus; and free of HCC at enrolment and during treatment. The patients included in this study were provided with appropriate and complete clinical information regarding the treatment regimen and duration. All the study participants provided informed consent. The study was conducted in accordance with the ethical principles stated in the Declaration of Helsinki and was approved by the Ethics Committee of the National Taiwan University Hospital, Kaohsiung Medical University Hospital, China Medical University Hospital, Chang Gung Memorial Hospital in Kaohsiung and Linkou, and Academia Sinica.

The patients infected with HCV genotype 1 or genotype non-1 received a combination of Peg-IFN and ribavirin therapy for 48 and 24 weeks, respectively. Serum HCV RNA test results at treatment initiation, end of treatment, and 24 weeks after the end of treatment were recorded. SVR was determined from the serum HCV RNA test results at 24 weeks after the end of treatment. Data regarding patient demographics (sex and birthdate), antiviral treatment (type of medication, treatment duration, and virological response), and presence of liver cirrhosis (ultrasonography or histological findings) were obtained. Virological data (HCV RNA levels and HCV genotype) and clinical laboratory findings, such as platelet count (10^9^/L) and haemoglobin (g/dL), aspartate aminotransferase [AST] (U/L), alanine aminotransferase [ALT] (U/L), and α-fetoprotein (ng/mL) levels, at baseline and 24 weeks after the end of treatment were recorded. The fibrosis-4 score (FIB-4) was used as a surrogate to estimate the amount of scarring in the liver, and it was calculated from the patients’ age, serum ALT (U/L) and AST (U/L) levels, and platelet count (10^9^/L)^20^. All clinical parameters were measured through standard laboratory techniques. Serum HCV RNA was measured by a commercialized sensitive assay with polymerase chain reaction using the COBAS TaqMan HCV test, v2.0 (Roche Diagnostics, Indianapolis, NJ, USA).

### Ascertainment of HCC

At enrolment, none of the participants in this treatment cohort had been diagnosed with HCC. In this cohort, the patients received regular health examinations and monitoring during and after the end of antiviral treatment. The medical record verification of incident HCC diagnoses was based on the following criteria: histological examinations and the detection of a positive lesion through at least two different imaging techniques (abdominal ultrasonography, angiography, or computed tomography) or through one imaging technique accompanied with an elevated serum α-fetoprotein level of ≥400 ng/mL^21^. In addition to active follow-up, newly developed HCC cases from January 1, 2004 to December 31, 2014 were detected through computerised linkage with the profiles from the National Cancer Registration in Taiwan via matching of the participants’ identification number and birthdate. To ensure complete ascertainment, a linkage with the national death certification database was also performed to identify cases of HCC death. The ascertainment of newly developed HCC was complete and accurate^4^.

### Statistical Analysis

The baseline characteristic profiles of patients with SVR or non-sustained virological response (NSVR) at the time of treatment initiation were compared using chi-squared tests. In this prospective study, we estimated the incidence of HCC from treatment responses. For each patient, the person-years of follow-up were calculated from the date of treatment initiation to either the date of HCC identification, the date of death, or December 31, 2014, whichever came first. The incidence rates of HCC per 1,000 person-years were calculated by dividing the number of newly developed HCC cases by person-years of follow-up. Kaplan–Meier method curves were used to depict cumulative risks for HCC according to SVR or NSVR throughout the follow-up years, and these data were compared with log-rank tests.

The treatment efficacy was evaluated by comparing the incidence of HCC between patients with SVR or NSVR. Cox proportional hazards models were used to examine the magnitude of HCC risk reduction in patients with SVR. Several clinical predictors at treatment initiation, including liver cirrhosis, platelet count levels, α-fetoprotein levels, and haemoglobin concentration, were considered in multivariate models. Hazard ratios (HRs) with 95% confidence intervals (CIs) were used to assess the magnitude of the associations between risk predictors and HCC. Statistical significance levels were determined by a two-sided p value of 0.05. The proportionality assumption of Cox models was examined, and the assumption was not violated. To evaluate the relevant predictors associated with HCC risk after treatment, additional Cox’s proportional hazards models were performed by stratifying patients according to their SVR status. The clinical predictors identified at the time of determining the SVR status were used for subsequent analyses.

The dose–response relationship between platelet count and α-fetoprotein levels and the risk of HCC, after adjustment for other risk predictors, were examined for statistical significance, with a test for trends. All analyses were performed using the SAS statistical software package (version 9.1; SAS Institute Inc., Cary, NC, USA).

## Results

### Baseline characteristics of SVR and NSVR patients

The clinical profiles of the 4639 patients at the initiation of antiviral treatment are shown in Table 1. A total of 3939 (85%) patients achieved SVR and 700 (15%) did not achieve SVR. Patients who achieved SVR tended to possess the following characteristics: younger age, male sex, HCV genotype non-1 infection, low HCV RNA levels at treatment, absence of cirrhosis, platelet count ≥100 10^3^/μL, low α-fetoprotein levels, and lower proportions of fibrosis (FIB-4 ≥3.25; p < 0.05).

**Table 1:** Baseline characteristics of study participants.

Determinants	Total (N = 4639) N (%)	NSVR (N = 700) N (%)	SVR (N = 3939) N (%)	*p*-value
Age				
mean ± SD	53.8 ± 10.0	56.1 ± 9.4	53.4 ± 10.0	<0.0001
Gender				
Female	2304 (49.7)	393 (56.1)	1911 (48.5)	0.0002
Male	2335 (50.3)	307 (43.9)	2028 (51.5)	
HCV genotype*				
Non-1	2108 (45.4)	132 (18.9)	1976 (50.2)	<0.0001
1	2531 (54.6)	568 (81.1)	1963 (49.8)	
Log HCV RNA (IU/mL)				
mean ± SD	5.7 ± 1.1	6.2 ± 0.8	5.7 ± 1.1	<0.0001
ALT (U/L)				
mean ± SD	131 ± 106.5	112.1 ± 74.4	134.3 ± 110.9	<0.0001
AST (U/L)				
mean ± SD	89.6 ± 64.1	87.1 ± 56.0	90 ± 65.4	0.2302
Hemoglobulin (g/dl)				
mean ± SD	14.2 ± 1.5	14.1 ± 1.5	14.3 ± 1.5	0.0105
Platelet count (10^3^/uL)				
mean ± SD	173.3 ± 61.9	164.4 ± 81.8	174.8 ± 57.8	0.0031
AFP (ng/mL)				
mean ± SD	15 ± 45.3	21.8 ± 43.9	13.9 ± 45.4	0.0002
FIB-4 ≥ 3.25	1279 (31.1)	233 (40.0)	1046 (29.7)	<0.0001
Cirrhosis	796 (17.3)	201 (28.9)	595 (15.3)	<0.0001

HCV: hepatitis C virus; ALT: alanine aminotransferase; AST: aspartate aminotransferase; FIB-4: fibrosis-4 score; AFP: α-fetoprotein; SVR: sustained virological response; NSVR: non-sustained virological response.

### Incidence of HCC with SVR and NSVR

A total of 233 incident HCC cases were identified after a total of 26,163 person-years of follow-up, and the incidence of HCC was 8.9 per 1000 person-years. Table 2 shows the incident HCC cases, the observed person-years, and the rate of HCC per 1000 person-years, stratified according to the patients’ SVR status. Of the 3939 patients with SVR, 155 developed HCC after 22,548 person-years of follow-up, indicating an incidence rate of 6.9 per 1000 person-years. In addition, of 700 patients with NSVR, 78 developed HCC, indicating an incidence rate of HCC was 21.6 per 1000 person-years. Among both SVR and NSVR patients, those with liver cirrhosis at study entry had a higher HCC incidence than did those without liver cirrhosis (p < 0.01).

**Table 2:** Incidence of HCC, stratified by treatment response and presence of liver cirrhosis at study entry.

	Total number	Number of HCC cases	Observed period (person-year)	Rate per 1000 person-year
Total	4639	233	26162.84	8.91
SVR	3939	155	22548.39	6.87
-LC	595	77	3460.95	22.25
-non-LC	3304	78	18822.74	4.14
NSVR	700	78	3614.44	21.58
-LC	201	51	1030.69	49.48
-non-LC	494	27	2557.23	10.56

HCC: hepatocellular carcinoma; SVR: sustained virological response; NSVR: non-sustained virological response; LC: liver cirrhosis; non-LC: non-liver cirrhosis

*40 SVR patients and 5 NSVR patients lack of cirrhosis information

### Cumulative risk of HCC with SVR and NSVR

**Figure 1:**
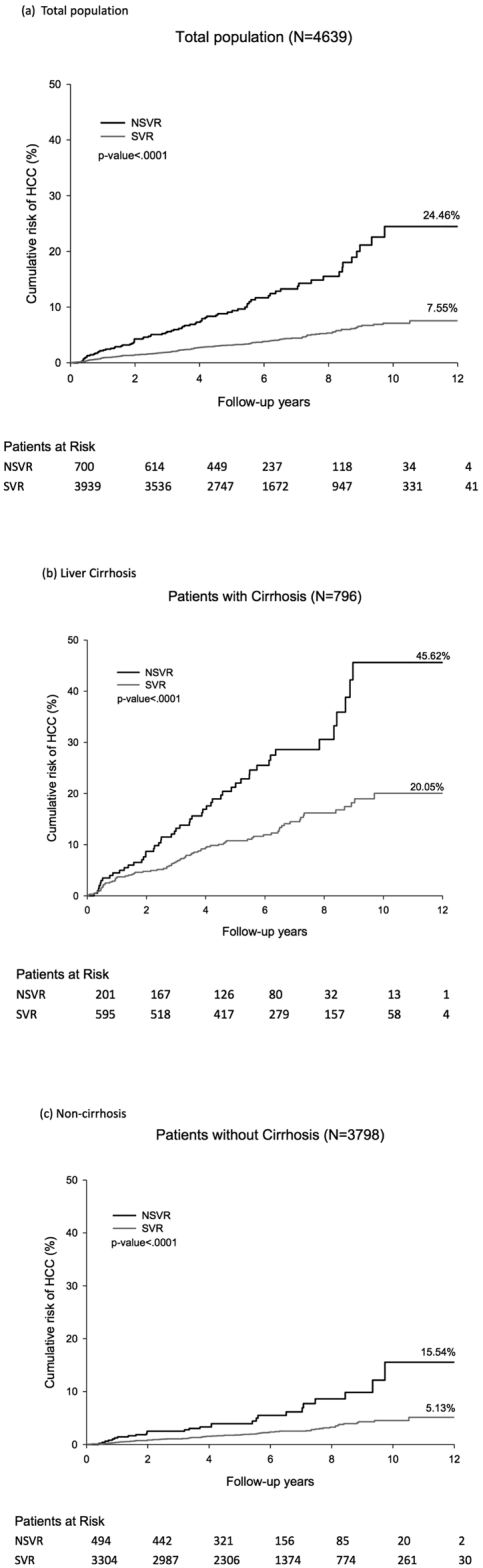
Cumulative risk of HCC after long-term follow-up. (**a**)Total participants. (**b**)Patients with liver cirrhosis at study entry. (**c**)Patients without liver cirrhosis at study entry.

The mean years of follow-up for the study patients was 5.6 years. The cumulative risk of HCC was 7.6% and 24.5% in patients with SVR and NSVR, respectively (p < 0.01; Fig. 1A). Patients with SVR had a decreased risk of HCC, irrespective of whether they had cirrhosis at study entry (p < 0.01). After follow-up, the cumulative risk of HCC was 20.1% for SVR and 45.6% for NSVR among cirrhotic patients and 5.1% for SVR and 15.5% for NSVR among non-cirrhotic patients.

### Treatment efficacy stratified by cirrhosis status at study entry

Among the non-cirrhotic patients, patients with SVR had a crude HR of 0.34 (0.20–0.57, p < 0.01) for HCC compared with those with NSVR, suggesting a treatment efficacy of 66%. Among patients with cirrhosis at study entry, the efficacy was 48%. These findings suggested that treating patients before they developed cirrhosis led to higher treatment efficacy.

### Pre-treatment seromarkers associated with HCC

Table 3 shows the relative risk of HCC, stratified by treatment response and other clinical predictors. In the univariate analysis, the development of HCC was associated with older age, elevated serum levels of AST and α-fetoprotein, low platelet count and haemoglobin levels, increased FIB-4, and the presence of liver cirrhosis (p < 0.01). After adjustment for the clinical predictors significantly associated with HCC at study entry, the adjusted HR (95% CI) was 0.44 (0.30–0.64) for patients with SVR compared with those with NSVR (p < 0.01). The associated risk of HCC in patients with SVR was 0.37 (0.22–0.63) for those without cirrhosis and 0.54 (0.31–0.92) for those with cirrhosis compared with their respective counterparts with NSVR.

**Table 3:** Adjusted hazard ratios and 95% confidence intervals of pretreatment predictors for HCC, stratified by presence of liver cirrhosis at study entry.

Determinants	Total patients Adjusted HR (95% CI)	Patients with liver cirrhosis Adjusted HR (95% CI)	Patients without liver cirrhosis Adjusted HR (95% CI)
Age	1.05 (1.03–1.08)	1.04 (1.01–1.07)	1.07 (1.04–1.10)
Gender			
Female	1.00	1.00	1.00
Male	2.51 (1.72–3.68)	2.78 (1.62–4.77)	1.86 (1.17–2.98)
Serum levels of AST (U/L)			
<45	1.00	1.00	1.00
45–89	0.81 (0.43–1.51)	0.88 (0.25–3.13)	0.85 (0.42–1.74)
≥90	1.13 (0.61–2.12)	1.48 (0.43–5.06)	0.99 (0.47–2.10)
p for trend	0.2324	0.1112	0.8453
Cirrhosis			
No	1.00	—	—
Yes	2.77 (1.93–3.99)		
Platelet count (10^3^/uL)			
≥200	1.00	1.00	1.00
100–200	1.54 (0.89–2.66)	1.20 (0.49–2.94)	1.81 (0.91–3.62)
<100	2.32 (1.25–4.32)	1.73 (0.71–4.27)	3.41 (1.40–8.30)
p for trend	0.0055	0.1189	0.0073
Alpha fetoprotein (ng/mL)			
<5	1.00	1.00	1.00
5–10	2.27 (1.35–3.82)	1.68 (0.65–4.32)	2.37 (1.27–4.43)
≥10	2.32 (1.35–3.99)	1.97 (0.80–4.90)	2.21 (1.11–4.41)
p for trend	0.0057	0.1523	0.0301
Hemoglobulin (g/dl)			
<13.6	1.00	1.00	
13.6–14.9	0.68 (0.46–1.01)	0.50 (0.28–0.89)	
≥14.9	0.41 (0.25–0.68)	0.22 (0.09–0.50)	
p for trend	0.0004	<0.0001	
Treatment			
NSVR	1.00	1.00	1.00
SVR	0.44 (0.30–0.64)	0.54 (0.31–0.92)	0.37 (0.22–0.63)

AST: aspartate aminotransferase; SVR: sustained virological response; NSVR: non-sustained virological response.

### Changes in seromarkers before and after treatment

A comparison of the seromarker levels before and after treatment showed significantly decreased (all p < 0.001) serum levels of ALT (mean ± SD = 131.0 ± 106.6 vs. 31.4 ± 34.1), AST (89.7 ± 64.2 vs. 31.9 ± 24.1), and α-fetoprotein (15.2 ± 46.4 vs. 5.4 ± 12.9). A total of 31.4% participants had FIB4 ≥ 3.25 before treatment, whereas 15.2% had FIB4 ≥ 3.25 after treatment (p < 0.001).

### Post-treatment seromarkers associated with HCC

The clinical predictors at 6 months after treatment cessation and the associated risk of HCC are described in Table 4. Among patients who achieved SVR, advanced age, male sex, elevated post-treatment AST levels, cirrhosis, decreased post-treatment platelet count, and increased post-treatment α-fetoprotein levels were significantly associated with HCC (p < 0.05). Moreover, advanced age, elevated post-treatment AST levels, and low post-treatment platelet count increased the risk of HCC among patients with NSVR (p < 0.05).

**Table 4:** Adjusted hazard ratios and 95% confidence intervals of post-treatment predictors for HCC, stratified by treatment response.

Predictors	Patients with SVR		Patients with NSVR	
	Total (95% CI)	Non-cirrhosis (95% CI)	Total (95% CI)	Non-cirrhosis (95% CI)
Age	1.08 (1.05–1.11)	1.1 (1.06–1.14)	1.07 (1.02–1.13)	1.06 (1.00–1.13)
Gender				
Female	1.00	1.00	1.00	1.00
Male	1.63 (1.03–2.58)	1.91 (1.04–3.51)	1.36 (0.61–3.03)	0.91 (0.29–2.83)
Serum levels of ALT (U/L)				
<45	1.00	1.00		
≥45	0.97 (0.45–2.1)	1.52 (0.52–4.47)		
Serum levels of AST (U/L)				
<45	1.00	1.00	1.00	1.00
≥45	2.47 (1.23–4.98)	1.79 (0.57–5.6)	6.20 (1.78–21.53)	4.11 (1.11–15.17)
Cirrhosis				
No	1.00	—	1.00	
Yes	2.14 (1.30–3.53)	—	1.01 (0.42–2.44)	
Platelet count (10^3^/uL)				
≥200	1.00	1.00	1.00	
100–200	1.30 (0.68–2.49)	1.54 (0.70–3.39)	1.00	
<100	2.19 (1.00–4.82)	1.92 (0.62–5.99)	5.52 (2.24–13.59)	
P for trend	0.0479	0.2225		
Alpha fetoprotein (ng/mL)				
<5	1.00	1.00		
5–10	1.25 (0.72–2.15)	1.35 (0.67–2.72)		
10–20	2.42 (1.16–5.08)	4.16 (1.45–11.97)		
≥20	9.58 (3.15–29.14)	8.54 (1.13–64.49)		
P for trend	0.0011	0.0053		

ALT: alanine aminotransferase; AST: aspartate aminotransferase; SVR: sustained virological response; NSVR: non-sustained virological response.

## Discussion

In Taiwan, a national viral hepatitis treatment programme was launched since October 2003. The government started reimbursing patients with chronic hepatitis B or hepatitis C for antiviral therapy. The incidence and mortality of end-stage liver diseases continually decreased in all age and sex groups from 2000–2003 (before the treatment programme) through 2004–2007 to 2008–2011. Using 2000–2003 as a reference, the incidence and mortality of HCC were decreased by at least 14–24%^22^. The availability of large national claims data and long-term follow-up enabled researchers to evaluate relatively rare outcomes such as HCC with sufficient power. This database has been utilised to evaluate the antiviral treatment efficacy, with results showing that patients who had received antiviral treatment had a decreased risk of liver-related disease incidence or mortality^22, 23^. However, the SVR status of the treated patients and the predictors for the development of HCC were difficult to assess because of the lack of individualised data regarding clinical markers. Our study enrolled a large number of patients with long-term follow-up and collected detailed clinical information, which helped to identify relevant seromarkers that could be useful for monitoring patients after treatment.

Although the nationwide Veterans Affairs HCV Clinical Case Registry contains health information for all-known HCV-infected patients and collects laboratory test as well as pharmacy data in a large cohort to examine SVR efficacy and liver-related diseases after considering for potentials confounders, this population was overwhelmingly male; thus, the results may not apply to populations with large proportions of females^15, 24^. The epidemiological characteristics of HCV infection and treatment responses in western and eastern countries were different; thus, a large follow-up study to estimate the treatment efficacy is warranted before the widespread use of the highly expensive new drugs^25^. A recent study recruited veterans with HCV infection and reported that HCC may still occur among those with SVR, at an incidence of 3.3 per 1000 person-years^24^. Although our cohort had more female patients, the estimated incidence of HCC was 6.9 per 1000 person-years, which was still higher than that among the veterans. In the veteran cohort, 64% of the participants were white, and that study revealed ethnic differences in the occurrence of HCC, with Asians having an approximately three-fold higher risk of developing HCC than do whites^24^. Hepatitis B virus infection is endemic to Taiwan and other Asian countries. In our study, all participants showed seronegativity for the hepatitis B surface antigen. Although we did not test antibodies against the hepatitis B core antigen, most patients who had ever been infected by hepatitis B virus were presumably in their early childhood. Recent meta-analysis demonstrated that the risk of HCC among anti-HCV-seropositives was significantly higher among the subject positive for anti-HBc than the subjects negative for anti-HBc^26^. The risk of anti-HBc-positivity on HCC occurrence remained even after successful antiviral therapy^27^.

Patients with SVR still had a substantially increased risk of developing HCC. This study emphasised the importance of monitoring, even for patients that had treatment-induced RNA clearance^28^ and particularly for those with fibrosis (low platelet count) and elevated serum α-fetoprotein levels. Patients without SVR had increased risk for HCC. Thus it will be essential to intensively monitor the patients who were failed for interferon-based regimen and allocate them with direct-acting antivirals.

This long-term follow-up study suggested that among patients with SVR, elevated serum α-fetoprotein levels after antiviral treatment were significantly associated with HCC risk in a dose–response-dependent manner. Serum α-fetoprotein levels are frequently used as a biomarker for HCC, but their performance was not satisfactory. When 20 ng/mL was used as the cutoff value for HCC detection, the sensitivity was 41–65% and specificity was 80–94%^29^. Patients with chronic viral hepatitis and cirrhosis in the absence of HCC may still have elevated α-fetoprotein levels^30^. Among chronic hepatitis C patients with advanced fibrosis, hepatic injury or regeneration may increase the serum α-fetoprotein levels even in patients without HCC^31, 32^. Elevated serum α-fetoprotein levels were associated with a decreased platelet count and an increased ALT-to-AST ratio^33^. The findings of our study were consistent with those of a Japanese cohort study, which showed that post-treatment serum α-fetoprotein levels achieved 80% predictability in detecting HCC by setting the cutoff value at 6 ng/mL^34^. Similarly, a recent study revealed that patients with α-fetoprotein levels higher than 10 ng/mL had an approximately 7.8-fold higher risk of developing HCC after achieving SVR^35^.

Regardless of the SVR status among patients, the discovery of biomarkers that may increase the predictive ability to identify patients with a high risk of developing HCC remains crucial. A prospective study in Japan found a unique fibrosis-related glycomarker that may predict the development of HCC with sufficient diagnostic accuracy^36^. Serum gamma-glutamyl transferase levels, a surrogate of oxidative stress, were associated with a subsequent HCC risk among noncirrhotic patients after HCV eradication^37^. These seromarkers were not routinely used in clinical settings but could be viewed as potential biomarkers for HCC surveillance. Both positive and negative predictive values of these biomarkers in conjunction with abdominal ultrasonography for liver surveillance should be evaluated. In the future, large-scale follow-up studies will facilitate the discovery of more useful biomarkers for end-stage liver disease.

HCV 1b and 2a were the predominant HCV genotypes in Taiwan. Previous studies have shown that patients with genotype 1 had an SVR rate of 70–80%^38–40^, whereas genotype 2 had an SVR rate of 74–90%^41, 42^. Compared with SVR rate in Western countries, the high SVR rate in Taiwan was due to a higher proportion of HCV genotype non-1 (40–45%) in Taiwan and a very high proportion of favorable IL28B genotype distribution (more than 90%) in Taiwanese HCV patients. Approximately 80–90% of East Asian people carried the favourable IL28B genotype, and the polymorphisms contributed to the treatment-induced RNA clearance and RNA spontaneous clearance^8, 43–45^.

In addition to hepatic diseases, chronic HCV infection was found to cause extrahepatic diseases^46–48^. However, less than 15% of chronic hepatitis C patients seek clinical care; thus, the community effectiveness of treatment is approximately 7–11%^49, 50^. Our study suggested that early intervention had higher efficacy for reducing HCC risk in hepatitis C patients without liver cirrhosis than in those with cirrhosis. The establishment of health policies for identifying asymptomatic HCV-infected patients living in the community through large-scale screening and recommending these patients for clinical care will be critical issues in the future. In addition, to reduce the patient risk of both hepatic and extrahepatic diseases, an increase in the patient accessibility to new effective drugs is essential. With regard to public health, the goal of primary prevention should be to promote an increased awareness and knowledge of hepatitis C through effective health education.

In conclusion, our findings suggest that treating chronic hepatitis C patients before the development of advanced fibrosis may result in improved clinical efficacy for decreasing the risk of HCC. In addition, patients with SVR still require continuous surveillance for HCC.
